# Wide local excision, Mohs micrographic surgery, and reconstructive options for treatment of dermatofibrosarcoma protuberans of the breast: A retrospective case series from Mayo Clinic

**DOI:** 10.1186/s12957-023-03022-9

**Published:** 2023-05-06

**Authors:** Hiba Saifuddin, Maria Yan, James Jakub, Jorys Martinez-Jorge, Randall Roenigk, Aparna Vijayasekaran

**Affiliations:** 1 Division of Plastic Surgery, Mayo Clinic, Rochester, MN USA; 2 Department of Surgery, Mayo Clinic, Jacksonville, FL USA; 3 Department of Dermatology, Mayo Clinic, Rochester, MN USA

**Keywords:** Dermatofibrosarcoma protuberans, Wide local excision, Mohs micrographic surgery, Reconstructive defect coverage, Primary closure

## Abstract

**Background:**

Dermatofibrosarcoma protuberans (DFSP) of the breast is a dermal fibroblastic neoplasm requiring wide excisional margins due to recurrence rates ranging from 26 to 60%. The current literature on reconstructive options and utility of Mohs micrographic surgery for DFSP of the breast is scarce. We describe surgical management of DFSP of the breast at our institution with the largest case series reported to date.

**Methods:**

A retrospective review was performed of women who underwent surgery for DFSP of the breast at our institution between 1990 and 2019. Continuous data was summarized using mean, median, and range; categorical data was summarized with frequency count and percentage. Preoperative lesion size and postoperative defect size were evaluated using 2-sided Fisher exact test, and *p*-values < 0.05 were considered statistically significant.

**Results:**

Nine patients underwent wide local excision (WLE) with reconstruction including pedicled latissimus dorsi flaps (*n* = 2), local flap advancement (*n* = 2), mastectomy with implant (*n* = 1), oncoplastic breast reduction (*n* = 1), and skin grafts (*n* = 3). Nine underwent Mohs micrographic surgery (MMS) with complex primary closure. Mean postoperative maximum wound defect size for WLE was 10.8 cm versus 7.0 cm for MMS with no statistical significance (*p* = 0.77). Mean preoperative maximum lesion size for WLE was 6.4 cm versus 3.3 cm for MMS with no statistical significance (*p* = 0.07). Complications with WLE included wound dehiscence in three patients and seroma in one patient. No complications were reported with MMS and primary closure. Recurrence was reported in one WLE patient, which was successfully detected despite flap coverage and resected without complications. Median follow-up for the patients without recurrence was 5.0 years, with two patients in MMS cohort lost to follow-up. Five-year overall survival was 100%.

**Conclusions:**

MMS and WLE are both viable surgical options for managing DFSP of the breast. MMS could potentially minimize reconstructive needs due to smaller average defect size and result in fewer complications but may also result in asymmetry. Immediate flap reconstruction, especially in larger defects, can achieve excellent aesthetic outcomes for patients with DFSP of the breast without compromising detection of disease recurrence.

## Background

Dermatofibrosarcoma protuberans (DFSP) is a rare neoplasm of dermal fibroblastic origin that comprises 1% of all soft tissue sarcomas [1]. It most frequently appears on the trunk and extremities but can also involve the breast [2]. Most patients are diagnosed between the 2nd and 4th decades of life [2]. Due to its appearance and slow growth often over many years, it is often undiagnosed or misdiagnosed as a cyst, dermatofibroma, or keloid [3]. Although it has a low incidence of metastasis and 5-year survival rate approaching 100%, it is locally aggressive with a recurrence rate of 0–30% with wide local excision (WLE) and 26–60% recurrence rate with narrow or positive margins [4-6]. This presents a challenge for balancing local control and cosmesis.

Current National Comprehensive Cancer Network (NCCN) guidelines for the treatment of DFSP recommend WLE with 2–4-cm gross margins or Mohs micrographic surgery (MMS) [7]. In the case of WLE, large defects can result following resection due to large margins, often requiring reconstructive procedures with flaps or skin grafts for coverage [6]. An additional challenge is balancing adequate resection for margin control and the aesthetic goals of symmetry, especially with breast lesions. The present literature on DFSP of the breast is scarce, with single institution case series describing 1–6 patients resected by WLE [8]. Although there are no established guidelines for breast reconstruction following DFSP resection, one case report of reconstruction following WLE recommended delaying reconstruction at least 2 years as it may prevent detection of local recurrence and will result in better aesthetic outcomes [3]. The aim of our study is to describe the surgical management, reconstructive procedures, and postoperative outcomes of 18 patients with DFSP of the breast resected by WLE and MMS at Mayo Clinic. We present the largest case series to date at a single institution.

## Materials and methods

Our study was exempt from Mayo Clinic IRB. We performed a retrospective review of medical records including women who were diagnosed with DFSP of the breast with a positive CD34 immunostain on biopsy. These patients underwent surgical resection and reconstruction at our institution between May 1990 and May 2020. We retrieved data such as sociodemographic variables, tumor location, type of resection, size of margins, recurrences, duration of follow-up, preoperative lesion size, postoperative defect size, type of closure or coverage, comorbidities, and possible complications such as partial or full flap necrosis, seroma, hematoma, dehiscence, or surgical revision. Continuous data was summarized using mean (standard deviation), median, and range; categorical data was summarized with frequency count and percentage. A lesion of the breast was defined as within the borders of the clavicle, midaxillary line, and inframammary fold. Preoperative lesion size and postoperative defect size were evaluated using 2-sided Fisher exact test, and *p*-values < 0.05 were considered statistically significant. The duration of follow-up was defined as date of tumor excision through date of last breast exam.

## Results

### Clinical characteristics

Between 1990 and 2020, a total of 18 women with DFSP of the breast were treated surgically at our institution. The average age at the time of surgery was 39.8 years (range 23–70), with a demographic composition of 72% (13/18) White, 11% (2/18) Arab, 11% (2/18) Hispanic, and 6% (1/18) Chinese. Most patients presented with concern for an enlarging unilateral lesion which varied from 1.5 to 6 cm on physical exam. Two patients had suspicious keloidal scars 12 cm in length. Six patients had a lesion in the upper outer quadrant, six patients in the upper inner quadrant, and six patients in the lower inner quadrant. Provisional diagnoses prior to excisional biopsy included epidermal inclusion cyst, sebaceous cyst, keloidal scar, myxoid neurofibroma, supernumerary nipple, and dermatofibrosarcoma. With provisional diagnosis in mind, 17/18 patients had an excisional biopsy and were diagnosed with DFSP by positive immunohistochemistry stain for CD34. Patients were then referred to our institution for definitive management as pathology showed either positive margins or incomplete resection of DFSP. One patient had prior recurrence of DFSP and underwent resection without preoperative biopsy. Two patients in the MMS cohort were lost to follow-up, and their missing data such as recurrence and complications were excluded from analysis.

### Treatment characteristics

**Fig. 1:**
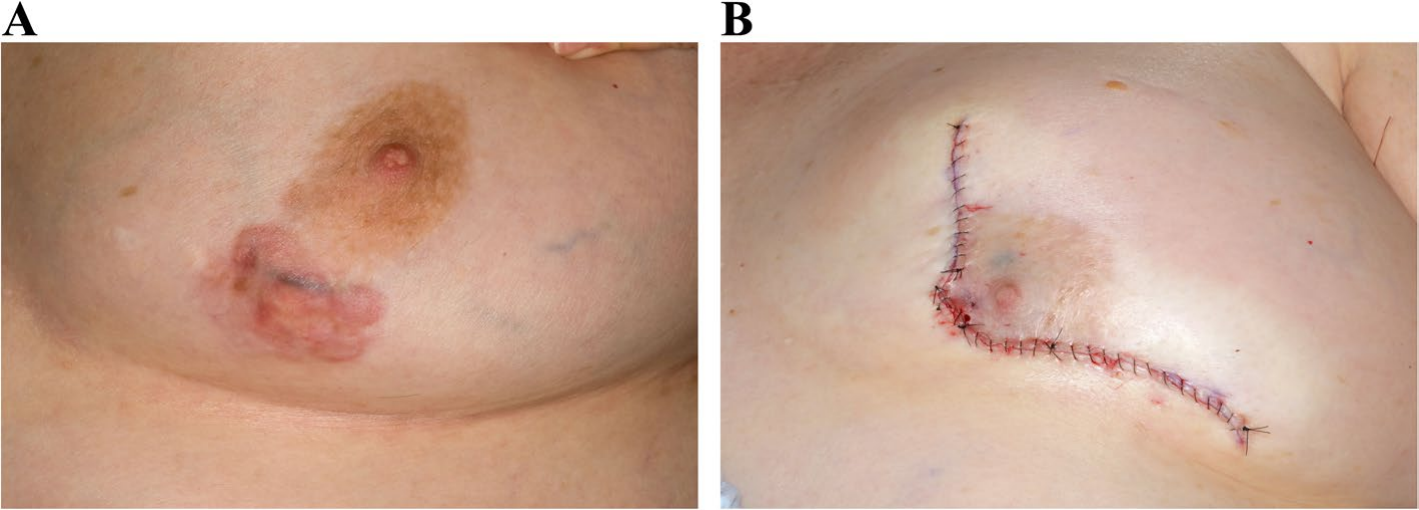
**A** DFSP lesion size of 6.8 cm in lower inner quadrant. **B** Immediate postoperative result of 6.8 cm lesion in lower inner quadrant treated by MMS and primary closure

**Fig. 2:**
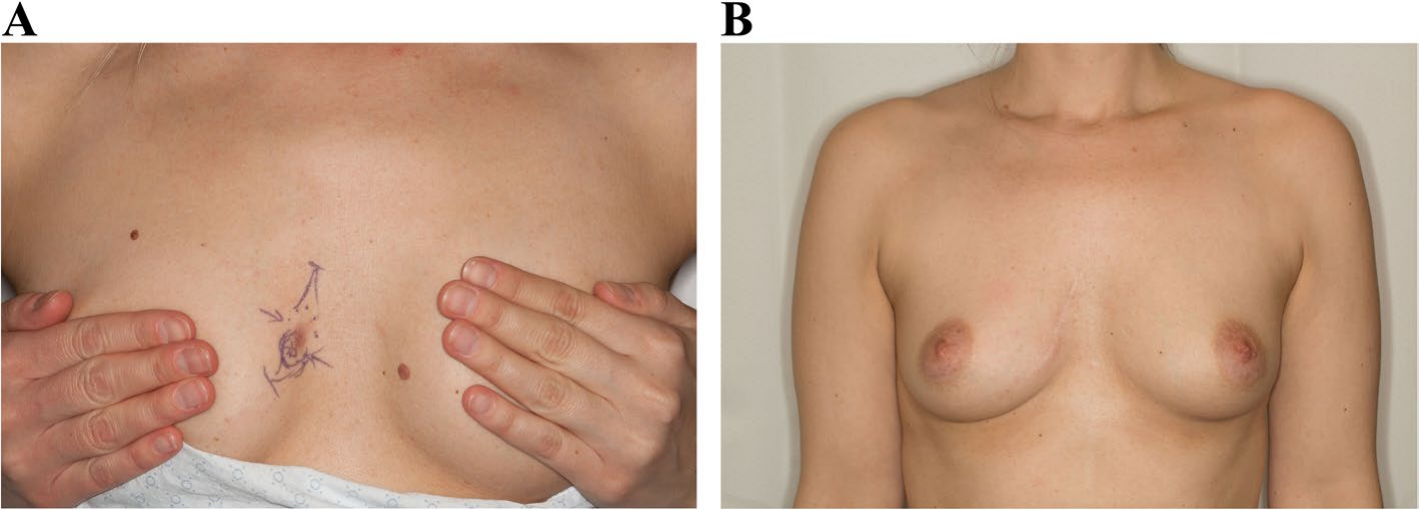
**A** DFSP lesion size of 2.7 cm in upper inner quadrant. **B** Postoperative result of 2.7-cm lesion in upper inner quadrant treated by MMS and primary closure after 5 years

**Fig. 3:**
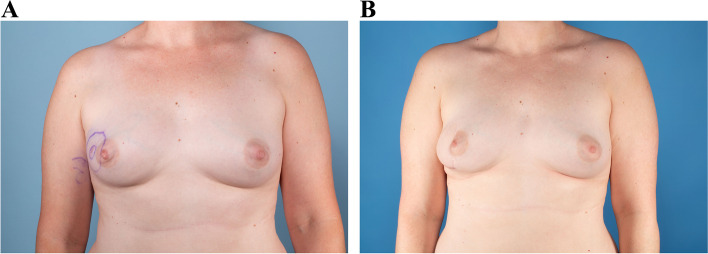
**A** DFSP lesion size of 3.5 cm in upper outer quadrant. **B** Postoperative result of 3.5-cm lesion in upper outer quadrant treated by WLE and coverage with pedicled latissimus dorsi flap after 17 months

**Fig. 4:**
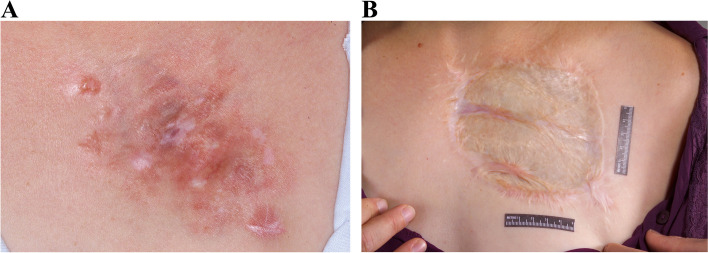
**A** DFSP lesion size of 12 cm in upper outer quadrant. **B** Postoperative result of 12-cm lesion in upper outer quadrant treated by WLE and coverage with skin graft after 4 years

Of the 18 women, 9 had DFSP resected by wide local excision (WLE) with margins determined by intraoperative pathologic frozen section analysis (Table 1). Nine patients were treated with Mohs surgery followed by complex primary closure (Table 2). Reconstruction for patients who underwent WLE included the following: latissimus dorsi flaps (*n* = 2), local flap advancement (*n* = 2), mastectomy with implant (*n* = 1), oncoplastic breast reduction (*n* = 1), and split-thickness skin grafts (*n* = 3). Three patients with flap and implant reconstruction had prior breast surgery including bilateral reduction mammoplasty, bilateral prepectoral silicone implants, and prior excision of DFSP with transverse rectus abdominis muscle flap (TRAM) reconstruction. Figures 1, 2, 3 and 4 illustrate the aesthetic results of MMS with primary closure and WLE with flap or skin graft coverage of varying lesion sizes in different breast quadrants.

**Table 1:** Patient characteristics for dermatofibrosarcoma protuberans breast lesions treated by WLE

**Age at surgery**	**Type of reconstruction**	**Quadrant location**	**Preoperative lesion size (cm)**	**Postoperative defect size (cm)**	**Complications and surgical interventions**	**Recurrence**	**Revision and reason**	**Closest microscopic margin (cm)**	**Intended gross margins (cm)**	**Number of intraoperative re-excisions**	**Prior surgeries**	**Follow-up**
62	Reverse abdominoplasty	Lower inner	5.5	12.5	None	None	None	1	3	0	None	14 years
30	Pedicled myocutaneous latissimus dorsi flap	Upper outer	6	8	None	Yes	None	0.9	1	1	Wide local excision with TRAM flap and 2-month chemotherapy without radiation	6 years
50	Local rotational flap	Lower inner	5.7	9.3	Seroma and superficial dehiscence at recipient site 32-day post-op aspirated and drain was placed	None	Excision of redundant IMF skin	1	N/A	0	Bilateral reduction mammoplasty and abdominoplasty	6 years
40	Pedicled myocutaneous latissimus dorsi flap	Upper outer	3.5	7.2	None	None	Scar revision, liposuction of lateral chest wall and standing cone secondary to contour abnormalities	1.7	2	1	None	1.4 years and ongoing
38	Two-stage implant-based reconstruction	Upper inner	2.5	Skin-sparing mastectomy	Recipient site full-thickness dehiscence 17-day postop surgically repaired	None	Implant exchange, bilateral capsulotomy with smooth round saline implant exchange secondary to marked distortion post tissue expansion	1	“Wide ellipse”	1	Bilateral prepectoral silicone implants	29 years
35	Pie crusted split-thickness skin graft	Upper outer	12	15	Recipient site superficial dehiscence 39-day postop treated with silver nitrate and Xeroform	None	None	1.7	N/A	1	None	16.3 years
35	Meshed split thickness skin graft	Upper inner	5	10	Recipient site mild skin breakdown and drainage at 4-month postop no intervention	None	None	0.6	N/A	1	None	4 years
55	Integra and full-thickness skin graft	Upper inner	12	17	None	None	None	1.5	N/A	1	None	25 months
35	Oncoplastic left breast reduction with contralateral breast reduction for symmetry	Lower inner	1.8	7.5	None	None	None	0.3	2	0	None	3 years and ongoing

**Table 2:** Patient characteristics for dermatofibrosarcoma protuberans breast lesions treated by MMS and primary closure

**Age at surgery**	**Quadrant location**	**Preoperative lesion size (cm)**	**Postoperative defect size (cm)**	**Complications and surgical interventions**	**Number of stages and blocks for clearance**	**Recurrence**	**Follow-up**
23	Upper inner	3.5	6.1	None	Debulking then 1 stage in 6 blocks	None	22.2 years
70	Lower inner	6.8	10.9	None	3 stages in 6/2/1 blocks	None	1 day
26	Upper outer	5	7	None	2 stages in 4/7 blocks	None	8.5 years
33	Upper inner	2.7	3.5	None	1 stage in 4 blocks	None	5.1 years
39	Upper inner	0.9	2.2	None	1 stage in 2 blocks	None	1 day
50	Upper outer	2.2	9.6	None	1 stage in 4 blocks	None	3.9 years and ongoing
30	Upper outer	1.2	6	None	3 stages in 4/1/2 blocks	None	5 years
39	Lower inner	3.8	12	None	3 stages in 6/4/2 blocks	None	3.1 years and ongoing
30	Lower inner	3.3	5.3	None	1 stage in 6 blocks	None	5 years

All flap reconstructions were performed the same day or within 2 days of WLE, and average microscopic closest margin for WLE cases was 1.1 cm (*SD* = 0.4). Gross intended margins available for 4/9 patients ranged from 1 to 3 cm. Six out of nine patients in the WLE cohort had intraoperative re-excision following positive margins on initial intraoperative pathology from skin-sparing mastectomy. Mean postoperative maximum wound defect size for WLE was 10.8 cm (range = 7.2–17 cm, *SD* = 3.7) with one patient undergoing skin-sparing mastectomy, while mean postoperative maximum wound defect size for MMS was 7.0 cm (range = 2.2–12, *SD* = 3.3) as shown in Table 3. Mean preoperative maximum lesion size for WLE was 6.4 cm (range = 1.8–12, *SD* = 3.7) and for MMS was 3.3 cm (range = 0.9–6.8 cm, *SD* = 1.8). There was no statistical significance in preoperative lesion size (*p* = 0.07) or postoperative defect size (*p* = 0.77) between MMS and WLE. One patient with prior history of DFSP recurrence who was reconstructed with a latissimus dorsi flap underwent 66 Gy of radiation postoperatively. No other patients received preoperative or postoperative chemotherapy or radiation.

**Table 3:** Comparing mean preoperative lesion size and postoperative defect size between MMS and WLE

	**Wide local excision** ***N* = 9**	**Mohs micrographic surgery** ***N* = 9**	***p*-value**
Preoperative maximum lesion size, cm			0.06
Mean (SD)	6.4 (3.7)	3.3 (1.8)	
Median (range)	5.6 (1.8–12)	3.3 (0.9–6.8)	
Postoperative maximum defect size, cm			0.6
Mean (SD)	10.8 (3.7)	7.0 (3.3)	
Median (range)	9.7 (7.2–17)	6.1 (2.2–12)	
Number of Mohs layers	-		
Mean (SD)	-	1.9 (0.9)	
Median (range)	-	2 (1–3)	
Recurrence	1	0	
Prior breast surgery	3	0	

In terms of complications, two of three patients with skin grafts had recipient site superficial wound dehiscence managed nonoperatively with dressings. The patient with two-stage implant reconstruction had recipient site full-thickness wound dehiscence requiring surgical intervention. One patient with local flap advancement had seroma formation requiring drainage. No complications were reported in the 7 patients with follow-up in the MMS cohort.

### Recurrence and follow-up

Local recurrence was reported in one patient treated with WLE (1/9) and flap at 6 months following surgery versus none in MMS cohort (0/7). This was resected with WLE and intraoperative frozen sectioning without complications. This patient also developed pulmonary metastases detected on CT scan at 9 months following excision of local recurrence. They were resected with no further recurrence of DFSP. Three patients reconstructed with a flap or implant underwent at least one revision surgery for redundant skin, contour abnormalities, or implant exchange. The median follow-up time was 5.0 years (Q1–3: 3.5–11.3), not including two patients who underwent MMS and lost to follow-up. Five-year overall survival was 100% for the 16 patients with follow-up.

## Discussion

We present a series of 18 patients with DFSP of the breast who underwent either MMS or WLE with either reconstructive surgery or primary closure. Our study showed a difference in lesion size of 3.3 cm in MMS versus 6.4 cm in WLE as well as excised specimen size of 7.0 cm in MMS compared with 10.8 cm in WLE. However, neither of these were statistically significant likely due to the small sample size. Although gross intended margins ranged from 1 to 3 cm with WLE in our series, about 67% (6/9) of patients with WLE had at least one intraoperative re-excision following positive margins on initial intraoperative pathology, indicating that the intended margins in these cases were not wide enough. Most DFSP recurrences are detected within 3 years of primary excision. Our study, with a median follow-up of 5 years, identified one patient with recurrence in the WLE cohort, but no recurrences with MMS [1]. Compared to primary closure, there was increased morbidity with more complex reconstructions, including flaps and grafts, but no surgical complications were observed in our two patients with pedicled flap coverage.

The major difference between MMS and WLE is the extent of resection of normal tissue and margin control [9]. A retrospective review of 48 patients with DFSP demonstrated more frequent positive margins in WLE than MMS, suggesting that MMS allows more focused resection resulting in accurate margin control [10]. Pathologic analysis of WLE specimens typically utilizes a vertical “breadloafing” technique, which can result in sampling error if the intervals of the sections miss extensions of tumor especially with DFSP’s infiltrative and asymmetric growth [9]. Most studies and systematic reviews report a lower recurrence rate with MMS compared to WLE [6, 11-20]. Similarly, studies utilizing modified WLE with total peripheral margin analysis and horizontal processing were able to achieve 0–1% recurrence rates, suggesting that meticulous margin evaluation is important regardless of surgical technique [5, 17, 21]. By focused excision of margins as directed by frozen section histologic review, MMS can also limit the size of postoperative defect compared to WLE [6, 7, 19, 22, 23]. Lowe et al. found a statistically significant smaller postoperative defect size by 2 cm in MMS compared to WLE in treatment of DFSP [6]. Goldberg et al. also found no recurrence in MMS despite MMS having average margin size of 1.36 cm compared to 2.33 cm for WLE [16]. This may be a deciding factor when resecting DFSP from a cosmetically sensitive area such as the breast, head, or neck [10, 21]. Similar to our study, there is also a trend of smaller lesions being more likely to be treated by MMS and larger lesions treated by WLE as DuBay et al., for example, reported in their study that preoperative lesions averaging 5.3 cm^2^ were treated by MMS, while preoperative lesions averaging 14.8 cm^2^ were treated by WLE [21].

All of our patients had unilateral DFSP of the breast, which can cause visible asymmetry following resection. There is clearly a size limit that will allow acceptable symmetry following primary closure with MMS, and this is also dependent on tumor location and breast size. For example, Fig. 1b shows loss of inferior pole with MMS and primary closure in a patient with a 6.8-cm lesion in the lower inner quadrant near the inframammary fold. Figure 2b demonstrates an excellent cosmetic result with MMS for a 2.7-cm lesion in the upper inner quadrant where there is less breast tissue. The use of a pedicled latissimus dorsi flap can preserve lower pole fullness and symmetry as seen in Fig. 3a and b, following resection of a 3.5-cm mass. MMS may be beneficial for patients who have a smaller lesion to breast size or location with less breast tissue, allowing for an aesthetically pleasing result with primary closure without further revision [24].

Interestingly for DFSP of the breast, there are no case reports that describe use of MMS for resection, very few case reports that describe reconstruction and its complications following resection, and no studies that compare complications between primary closure and reconstruction [8]. In addition to primary closure, reconstruction techniques cited in the literature include pedicled latissimus dorsi flap, rotation flap, reverse abdominoplasty, pectoralis flap, and reduction mammoplasty to provide wound coverage and to preserve breast shape [25-30]. We found no complications in our MMS and primary closure cohort but encountered complications requiring intervention with WLE and skin grafts, implant, and rotational flap reconstructions. One recurrence in the WLE cohort was detected despite pedicled flap reconstruction and resected without complications. Despite potential asymmetry, there still may be value to primary closure after MMS in select patients as it involves fewer complications, allows for time to monitor the wound for possible recurrence, and provides the patient more time to decide whether a simple repair is sufficient or a more complex delayed revision is desired. Although our flap reconstructions were immediate due to availability of intraoperative frozen sectioning, this may not translate to other institutions where frozen section is not available. All of these considerations should be discussed in shared decision-making with the patient when selecting among WLE, MMS, and timing of reconstruction.

A limitation of our retrospective study is likely selection bias in which patients were referred for WLE or MMS. Potentially, patients with larger tumors, smaller breast size, and/or those who voiced concern over cosmetics were referred to a plastic surgeon. There can be confounding factors such as surgeon preference or if patients were offered a plastic surgery consult to discuss possible elective reconstruction. Another limitation is we did not collect patient-reported outcomes, and thus, it is unknown if patients were satisfied with the aesthetic outcomes of the different techniques.

## Conclusion

This study is the largest case series of DFSP of the breast and describes two cohorts of patients managed by different resection modalities, reconstructions, and outcomes, which has been missing from the literature. Both MMS and WLE are viable options for resection, and we cannot conclude if one is superior. With either method, meticulous surgical margins and histologic analysis are important to minimize recurrence and allow for immediate reconstruction when required. Characteristics such as preoperative lesion size, postoperative defect size, and soft tissue availability can help determine if a simpler repair with MMS would be beneficial or a more complex staged reconstruction with WLE is preferred. Shared decision-making with the patient should also play a role in determining immediate reconstruction with WLE, primary closure with MMS, or staged delayed reconstruction with MMS.

## Data Availability

All data generated or analyzed during this study are included in this published article, and supplementary information is available from corresponding author on reasonable request.

## Abbreviations

DFSP: Dermatofibrosarcoma protuberans
WLE: Wide local excision
MMS: Mohs micrographic surgery
NCCN: National Comprehensive Cancer Network
